# Systemic regulation of soybean nodulation and nitrogen fixation by nitrogen via isoflavones

**DOI:** 10.3389/fpls.2022.968496

**Published:** 2022-08-11

**Authors:** Xiaochen Lyu, Chunyan Sun, Tao Lin, Xuelai Wang, Sha Li, Shuhong Zhao, Zhenping Gong, Ziwei Wei, Chao Yan, Chunmei Ma

**Affiliations:** ^1^College of Agriculture, Northeast Agricultural University, Harbin, China; ^2^College of Resources and Environment, Northeast Agricultural University, Harbin, China; ^3^College of Engineering, Northeast Agricultural University, Harbin, China; ^4^Harbin Agricultural Technology Extension Station, Harbin, China

**Keywords:** dual-root soybean, nitrogen, systemic regulation, isoflavones, nodulation

## Abstract

Nitrogen (N) inhibits soybean (*Glycine max* L.) nodulation and N_2_ fixation. Isoflavones secreted by soybean roots can stimulate signal transduction for symbiotic nodules, thus playing a key role in root nodule development and N_2_ fixation. The relationship between the inhibition of soybean nodulation, N_2_ fixation and isoflavones by N is still unclear. In this study, dual-root soybean plants were prepared by grafting, and N or isoflavones were supplied to unilateral roots. The number and dry weight of the soybean nodules, nitrogenase activity, isoflavone concentrations and relative changes in the level of expression of nodulation-related genes were measured to study the response relationship between the N systemic regulation the soybean nodule N_2_ fixation and changes in the concentrations of isoflavones in its roots. The results showed that N supply to one side of the dual-root soybeans systematically affected the N_2_ fixation of root nodules on both sides, and this effect began in the early stage of nodulation. Moreover, a unilateral supply of N systematically affected the concentrations of daidzein and genistein on both sides of the roots. The concentrations of isoflavones were consistent with the change trend of soybean root nodule and nodulation-related gene expression level. Treatment with unilateral N or isoflavones affected the soybean nodule N_2_ fixation and its nodulation-related genes, which had the same response to the changes in concentrations of root isoflavones. N regulates soybean nodulation and N_2_ fixation by systematically affecting the concentrations of isoflavones in the roots.

## Introduction

A symbiotic relationship is established between soybean (*Glycine max* L.) and rhizobium to form root nodules, which can fix N from the air for their own growth (Mulder et al., 2005). When the soybean root is exposed to a high concentration of N, nodulation and N_2_ fixation capacity will be inhibited; aging of the root nodules will be accelerated, and the N_2_ fixation efficiency will be reduced (Harper and Gibson, 1984; Carroll et al., 1985; Gibson and Harper, 1985; Streeter and Wong, 1988; Mizukoshi et al., 1995; Fujikake et al., 2003). Root hair deformation caused by the infection of leguminous crops by root nodulating bacteria is the most significant response during the early stage of nodulation (Zaat et al., 1987). *NFR1* and *NIN* are involved in the early nodulation processes, such as the identification of nodulation factors and infection line formation (Schauser et al., 1999; Indrasumunar et al., 2011). Abiotic stress suppresses the deformation of soybean root hairs while reducing the quantity of root nodules (Duzan et al., 2004). It has been demonstrated that a high concentration of N significantly reduces the rate of root hair deformation of Lotus japonicus and reduces the level of expression of *NIN*, while the level of expression in Lotus japonicus mutants resistant to high N remains at a high level (Barbulova et al., 2007). The overexpression of *GmNFR1* increased the number of nodules in soybean, and silencing *GmNFR1* inhibited the deformation of root hairs and the formation of infection threads in soybean (Indrasumunar et al., 2011). The early nodulin gene (Nguyen et al., 2019) is involved in the growth process of root nodules. After nitrate was applied to Medicago truncatula, the level of expression of the nodulin gene *ENOD93* was downregulated (Cabeza et al., 2014). Inoculating efficient rhizobia and treatment with 5 mM of nitrate increased the nodule number and dry weight of soybean, and the relative level of expression of *GmENOD40* also increased significantly (Nguyen et al., 2019). High amounts of N were supplied to the non-nodulation side of the unilateral nodulation dual-root soybean system, and the nodule number, dry weight and nitrogenase activity on the unsupplied nitrogen side were inhibited (Lyu et al., 2020; Lyu et al., 2022). Previous studies have suggested that the effect of N on N_2_ fixation by root nodules not only causes local inhibition but also systemic regulation (Hinson, 1975; Tanaka et al., 1985; Silsbury et al., 1986; Arnone et al., 1994; Yashima et al., 2003, 2005).

As important signal molecules in root nodule symbiosis, flavonoids can stimulate the synthesis and secretion of nodulation factors by rhizobium, stimulate the symbiotic signal transduction for nodulation, participate in root nodule organogenesis and regulate the formation of root nodules (Redmond et al., 1986). It is very important for rhizobium to establish a symbiotic relationship with leguminous crops. Flavonoid synthesis genes are induced during the early stage of nodulation. It has been demonstrated that bradyrhizobium induces the expression of genes for key enzymes, such as phenylalanine ammonia lyase (*PAL*) and chalcone synthetase (*CHS*), that are involved in flavonoid synthesis in soybean roots (Estabrook and Sengupta-Gopalan, 1991). RNAi interference technology was used to interfere and silence the gene for the rate-limiting enzyme (*CHS*) of the flavonoid synthesis pathway in *Medicago truncatula*, the concentrations of root flavonoids decreased and inhibited the formation of root nodules, while the exogenous supply of naringenin and liquiritigenin, the precursors of flavonoids, restored the normal nodulation and root flavonoid content of *Medicago truncatula* (Wasson et al., 2006). Daidzein and genistein are the primary isoflavones in soybeans. Most of the isoflavones in root system secretions during the early growth stage of soybean are daidzein derivatives, and genistein can change the composition and molecular weight distribution of the extracellular polysaccharides produced by rhizobium and plays a key role in the generation of nodules and the adjustment of nodule ratio (Sugiyama et al., 2016). Isoflavone-sensitive rhizobium can still identify the soybean root and nodulate normally when there are low concentrations of daidzein and genistein in soybean roots (Subramanian et al., 2006). Supplying N directly to the root system significantly reduced the concentrations of daidzein and genistein in soybean roots (Cho and Harper, 1991; Sugiyama et al., 2016). N systematically regulates soybean nodule N_2_ fixation whether isoflavones are involved merits further study.

In this study, we proposed the hypothesis that systemic effect of N on N_2_ fixation in soybean nodules is regulated by isoflavones. Thus, the dual-root soybean materials were prepared by grafting, and N or isoflavones were supplied to unilateral roots. The nodule number, nodule dry weight, nitrogenase activity, root isoflavone concentrations and changes in the level of expression of nodulation-related genes were measured to study the response relationship between the N systemic regulation of the soybean nodule N_2_ fixation and changes in the concentrations of isoflavones in its roots, so as to provide a basis and insights for the physiological regulatory mechanism of N_2_ fixation by nodulation in soybean.

## Materials and methods

The dual-root soybean material was prepared using the seedling grafting method of Xia et al. (2017), and a sand culture experiment was conducted. The soybean variety used was Heinong 40, and the N source tested was ammonium nitrate (NH_4_NO_3_). Two seedlings were grafted when the cotyledons of the soybean grew to 7–10 cm from the root tip. After 7 days of growth (cotyledon (VC) stage), the part above the grafted interface of downward notched seedlings was cut off, and two roots and one aboveground soybean seedling were prepared. They were then cultured and tested in the field. Supplementary material 1 shows the specific grafting method, preparation of nutrient solution and method of inoculating rhizobia.

### Experimental treatments

#### Experiment 1

Dual-root soybean was treated in the VC stage, and both sides of the N0 treatment were supplied with N-free nutrient solution. The N100 treatment N-supply side (N+) was supplied with a 100 mg/l N concentration of nutrient solution, and the N-free side (N−) was supplied with N-free nutrient solution. Samples were taken on 1, 3, 7, and 21 days after treatment. After 1 and 3 days of treatment, the number of deformed root hairs was measured by sampling. The root system on both sides was washed with distilled water to remove the sand, and the root system was observed by optical microscopy. The degree of root hair deformation was determined by the number of deformations in every 60 root hairs in the microscope field of vision, and each treatment was repeated six times. After 3 and 7 days of treatment, changes in the expression of genes in the roots were measured by sampling, and the expression of gene changes in the nodules was measured after 21 days of treatment. The root system on both sides was washed with distilled water, the nodules were then removed, and the roots were cut into pieces. The root and nodules were stored at −80°C to measure the level of gene expression, and each treatment was repeated three times. For RNA extraction and quantitative real-time reverse transcription-PCR (qRT-PCR) analysis, total RNA was extracted from root nodules using the TRIzol reagent (Servicebio, Wuhan, China), and cDNA was synthesized using a RevertAid reverse transcription kit (Servicebio, Wuhan, China). The primer sequences used for qRT-PCR amplification are shown in Supplementary material 2. Three biological replicates were performed. In this study, 18S rRNA was used as the reference to calculate the qRT-PCR data for genes as described by Carter and Tegeder (2016).

#### Experiment 2

The dual-root soybean was treated during the VC stage. The experiment was divided into two consecutive stages. The N_0_ treatment supplied N-free nutrient solution on both sides of the two stages. The N_100_ treatment supplied a nutrient solution with an N concentration of 100 mg/l on the N+ side of the two stages, and the N-side was supplied with N-free nutrient solution. In N_100-0_ treatment, the N+ side was supplied with nutrient solution with a concentration of 100 mg/l N in stage I and N-free nutrient solution in stage II. The N-side was supplied with N-free nutrient solution in two stages. There were two sampling times: (1) Stage I was treated for 21 days and stage II treatment was initiated at 22 days and ended at 42 days. Samples were taken on days 21 and 42, and the nodule number, nodule dry weight, and nitrogenase activity were determined (Lyu et al., 2019). Each treatment was repeated four times. (2) Stage I was treated for 7 days and stage II was started at 8 days and ended at 14 days. After 7 days of treatment, the soybean plants were completely pulled out. The roots were rinsed with distilled water and then transferred to a beaker that contained sterile deionized water, and the root exudates were collected for 48 h. The deionized water that contained the root exudates was passed through a 0.45 μm filter membrane, concentrated into a powder in a freeze-vacuum desiccator (Marin Christ, Osterode, Sachsen, Germany), and stored in a sterile desiccator for later use. Root samples were taken on days 7 and 14. The concentrations of daidzein and genistein in roots and root exudates were determined by high-pressure liquid chromatography (HPLC). Each treatment included four replicates.

#### Experiment 3

Isoflavones were unilaterally applied to a dual-root soybean during the VC stage. In the I_3.75_ treatment, the treated side (T+) of the roots was supplied with nutrient solution that contained isoflavone, and the concentrations of daidzein and genistein were 3.75 mg/l. The untreated side (T-) of the roots was supplied with the nutrient solution without isoflavone. The treatment without isoflavone nutrient solution on both sides was used as a control, which was recorded as I_0_. The N concentration of nutrient solution supplied on both sides of roots in two treatments was 25 mg/l. On day 7 of treatment, samples were taken to determine the concentrations of daidzein and genistein in roots and root exudates, and there were four replicates. On day 21 of treatment, samples were taken to determine the nodule number, nodule dry weight, and nitrogenase activity, and there were four replicates. The change in level of expression of genes in the roots was measured on days 3 and 7 of treatment, and the levels of expression of changes in the genes in nodules were measured 21 days after treatment. Each treatment was repeated four times. The specific sampling method and determination method were the same as those used in Experiments 1 and 2.

### Data analysis

Data processing and statistical analysis were performed with Microsoft Excel 2016 (Redmond, WA, USA) and SPSS 21.0 (IBM, Inc., Armonk, NY, USA). All the data were tested for normality before a one-way analysis of variance (ANOVA) was conducted. The mean differences were compared using Duncan’s multiple range test. Comparisons with *p* < 0.05 were considered significant.

## Results

### Nodulation and nitrogenase activity in dual-root soybeans

**Figure 1:**
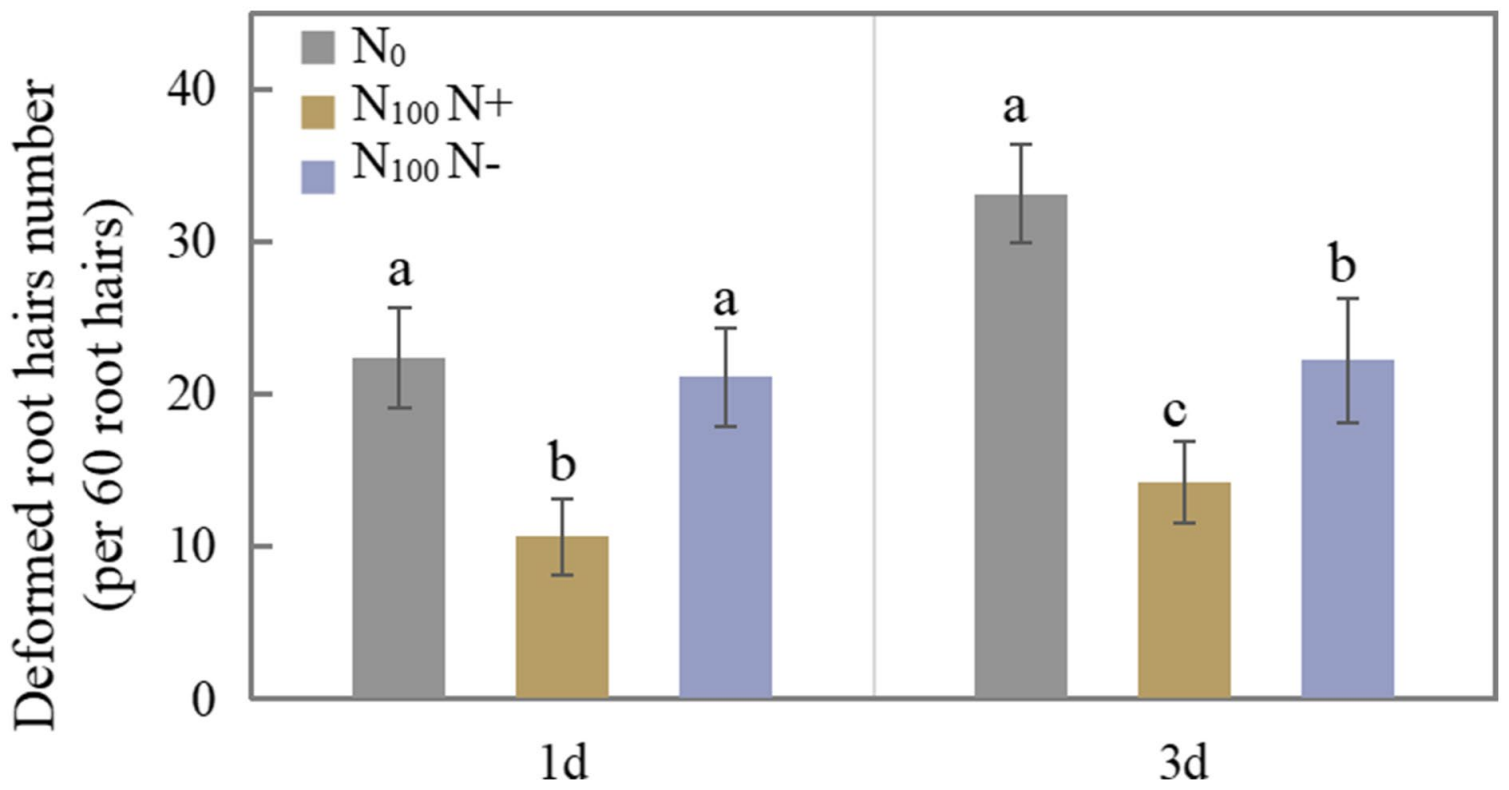
Number of deformed root hairs of dual-root soybeans, N_0_ is N-free nutrient solution on both sides of dual-root soybeans, N_100_ N+ is N-supply side of dual-root soybeans treated with 100 mg/l nutrient solution on unilateral side, N_100_ N-is N-free side of dual-root soybeans treated with 100 mg/l nutrient solution on unilateral side, different lowercase letters above the bars indicate significant difference (*p* < 0.05) by Duncan’s multiple range test.

Figure 1 shows the difference in the number of deformed root hairs after N supply for 1 and 3 days. The number of deformed root hairs on the N+ side of N100 treatment for 1 day was 52.50% lower than that of the N0 treatment. The number of deformed root hairs on the N-side treated with N100 did not differ significantly from that treated with N0. The N supply treatment inhibited root hair deformation on both sides of the soybean for 3 days. The number of deformed root hairs on the N+ side of N100 treatment was 57.10% lower than that of the N0 treatment, and the number of deformed root hairs on the N-side of N100 treatment was 32.94% lower than that of the N0 treatment, indicating that N supply inhibited root hair deformation and thus affected nodulation. With the increase in time of N supplied, the degree of inhibition of root hair deformation on both sides of the dual-root soybean intensified, indicating that the N supply hindered the growth of root nodules on the direct contact side, while the growth of root nodules without contacting an N source also tended to be inhibited.

Table 1 shows the changes of nodules on the dual-root soybeans. After 21 days of N supply treatment, the nodule number and dry weight of the N-side in N_0_ and N_100_ treatments were similar. Compared with the N_0_ treatment, the nodule number on the N+ side of N_100_ treatment decreased by 38.14%, and nodule dry weight decreased by 36.84%. After 42 days of N supply, the nodule number and dry weight of the N+ side were significantly different among the three treatments. Compared with the N_0_ treatment, the nodule number decreased by 26.32 and 17.73% in the N_100_ and N_100-0_ treatments, respectively, and nodule dry weight decreased by 30.61 and 14.29%, respectively. The N_100_ and N_100-0_ treatments were close to the number and dry weight of the N-side nodules. In comparison with the N_0_ treatment, nodule dry weight was reduced by 14.29 and 12.24%, and nodule number was reduced by 11.57 and 9.71%, respectively. This indicated that N supply hindered the growth of nodules on the direct contact side, and the growth of nodules without N was also inhibited. The removal of N from the N-supply roots restored the growth of nodules.

**Table 1:** Changes of dual-root soybean nodules.

	Treatments	21 days		42 days	
		N+	N−	N+	N−
Nodule number (per plant)	N_0_	104.0 ± 9.71a	104.0 ± 9.71a	160.7 ± 8.72a	160.7 ± 8.72a
	N_100_	64.3 ± 6.73b	91.3 ± 6.17a	118.4 ± 8.01c	142.1 ± 7.31b
	N_100-0_			132.2 ± 6.14b	145.1 ± 5.14b
Nodule dry weight (g/plant)	N_0_	0.19 ± 0.033a	0.19 ± 0.033a	0.49 ± 0.041a	0.49 ± 0.041a
	N_100_	0.12 ± 0.019b	0.18 ± 0.061a	0.34 ± 0.022c	0.42 ± 0.062b
	N_100-0_			0.42 ± 0.031b	0.43 ± 0.021b

N_0_ is the N-free nutrient solution treatment on both sides of the dual-root soybeans, N_100_ is the N-supply concentration of 100 mg/l nutrient solution on the unilateral side of the dual-root soybeans, and N_100-0_ is the N-supply concentration of 100 mg/l on the unilateral side of the dual-root soybeans and it was changed to N-free nutrient solution. N+ is the N-supply side, N-is the N-free side, the values in the table represent the mean ± standard error (*n* = 4), and different lowercase letters indicate significant difference (*p* < 0.05) by Duncan’s multiple range test and longitudinal comparison.

As shown in Table 2, changes in the specific nitrogenase activity (SNA) and acetylene reduction assay (ARA) of the dual-root soybean nodules were measured. After 21 days of unilateral N supply, the SNA and ARA of N+ side of N_100_ treatment were significantly lower than those of N_0_ treatment, which decreased by 24.77 and 52.58%, respectively. There was no significant difference between N-side SNA and ARA of N_100_ and N_0_ treatments. After 42 days of unilateral N supply, SNA and ARA of N+ side of each treatment were significantly different. Compared with N_0_ treatment, SNA of N_100_ and N_100-0_ treatments decreased by 38.71 and 14.31%, respectively, and that of ARA decreased by 57.45 and 26.54%, respectively. The N-side SNA and ARA of N_100_ and N_100 0_ treatments were very similar, and the SNA of the two treatments decreased by 21.17 and 14.92% compared with N_0_ treatment, while the ARA decreased by 34.43 and 25.31% compared with N_0_ treatment, respectively. This indicated that the N supply inhibited the nitrogenase activity of the nodules, and the nitrogenase activity was restored after N-free culture. Changes in the N-side were smaller than those in the N+ side.

**Table 2:** Changes of nitrogenase activity in dual-root soybean nodules.

	Treatments	21 days		42 days	
		N+	N−	N+	N−
SNA (C_2_H_4_ μmol g^−1^ nodule dry mass h^−1^)	N_0_	22.4 ± 2.91a	22.4 ± 2.91a	49.6 ± 3.61a	49.6 ± 3.61a
	N_100_	16.8 ± 0.41b	21.2 ± 2.90a	30.4 ± 1.87c	39.1 ± 2.72b
	N_100-0_			42.5 ± 1.87b	42.2 ± 1.65b
ARA (C_2_H_4_ μmol h^−1^ plant^−1^)	N_0_	4.2 ± 0.07a	4.2 ± 0.07a	24.3 ± 1.74a	24.3 ± 1.74a
	N_100_	2.0 ± 0.22b	3.8 ± 0.04a	10.3 ± 0.98c	16.4 ± 1.47b
	N_100-0_			17.8 ± 2.41b	18.1 ± 1.12b

N_0_ is the N-free nutrient solution treatment on both sides of the dual-root soybeans, N_100_ is the N-supply concentration of 100 mg/l nutrient solution on the unilateral side of the dual-root soybeans, and N_100-0_ is the N-supply concentration of 100 mg/l on the unilateral side of the dual-root soybeans and it was changed to N-free nutrient solution. N+ is the N-supply side, N-is the N-free side, the values in the table represent the mean ± standard error (*n* = 4), and different lowercase letters indicate significant difference (*p* < 0.05) by Duncan’s multiple range test and longitudinal comparison.

### Isoflavones concentrations in dual-root soybean roots and root exudates

Table 3 shows the changes of daidzein and genistein concentrations in dual-root soybean roots. After 7 days of N supply, the daidzein concentration in N+ side roots of N_100_ treatment decreased by 27.95% and N-side by 13.90% compared with N_0_ treatment. The genistein concentration in N+ side root of N_100_ treatment decreased by 40.61% compared with N_0_ treatment, and genistein concentration in N-side decreased by 20.30%. After 7 days of unilateral N supply, the concentrations of daidzein and genistein in the root exudates of the N+ side in the N_100_ treatment were significantly lower than those in the N_0_ treatment, while those in the N-side were also smaller than those in the N_0_ treatment, but the difference was not significant (Supplementary Table S3). The changes of daidzein and genistein concentrations in root exudates of the dual-root soybeans were consistent with those in roots. After 14 days of nitrogen supply treatment, the daidzein concentrations in N+ side roots of each treatment were significantly different, and the daidzein concentrations in N_100_ and N_100-0_ treatments were 23.75 and 17.12% lower than those in N_0_ treatment, respectively. The daidzein concentration in N-side roots of N_100_ and N_100-0_ treatments was also significantly lower than those of N_0_ treatment, which decreased by 18.86 and 11.90%, respectively. The genistein concentration in N+ side of N_100_ and N_100-0_ treatment decreased by 37.46 and 27.84% compared with N_0_ treatment. The genistein concentration in N-side of N_100_ and N_100-0_ treatments decreased by 22.68 and 19.59% compared with N_0_ treatment. This indicated that a supply of N inhibited the concentrations of root daidzein and genistein on both sides of the dual-root soybean. In addition, the removal of N from the treatment that had been supplied with high N increased the concentrations of daidzein and genistein on both sides of the dual-root soybean, and the change was more significant on the N+ side.

**Table 3:** Concentrations of daidzein and genistein in dual-root soybean roots (mg/g DW).

	Treatments	7 days		14 days	
		N+	N−	N+	N−
Daidzein	N_0_	3.86 ± 0.031a	3.86 ± 0.035a	4.16 ± 0.034a	4.16 ± 0.035a
	N_100_	2.78 ± 0.033b	3.32 ± 0.013b	3.17 ± 0.084c	3.38 ± 0.142b
	N_100-0_			3.45 ± 0.075b	3.67 ± 0.051b
Genistein	N_0_	0.197 ± 0.003a	0.197 ± 0.003a	0.291 ± 0.010a	0.291 ± 0.010a
	N_100_	0.117 ± 0.027b	0.157 ± 0.006a	0.182 ± 0.003c	0.225 ± 0.002b
	N_100-0_			0.210 ± 0.001b	0.234 ± 0.002b

N_0_ is the N-free nutrient solution treatment on both sides of the dual-root soybeans, N_100_ is the N-supply concentration of 100 mg/l nutrient solution on the unilateral side of the dual-root soybeans, and N_100-0_ is the N-supply concentration of 100 mg/l on the unilateral side of the dual-root soybeans and it was changed to N-free nutrient solution. N+ is the N-supply side, N-is the N-free side, the values in the table represent the mean ± standard error (*n* = 4), and different lowercase letters indicate significant difference (*p* < 0.05) by Duncan’s multiple range test and longitudinal comparison.

### Relative expression of nodulation-related genes in dual-root soybeans

**Figure 2:**
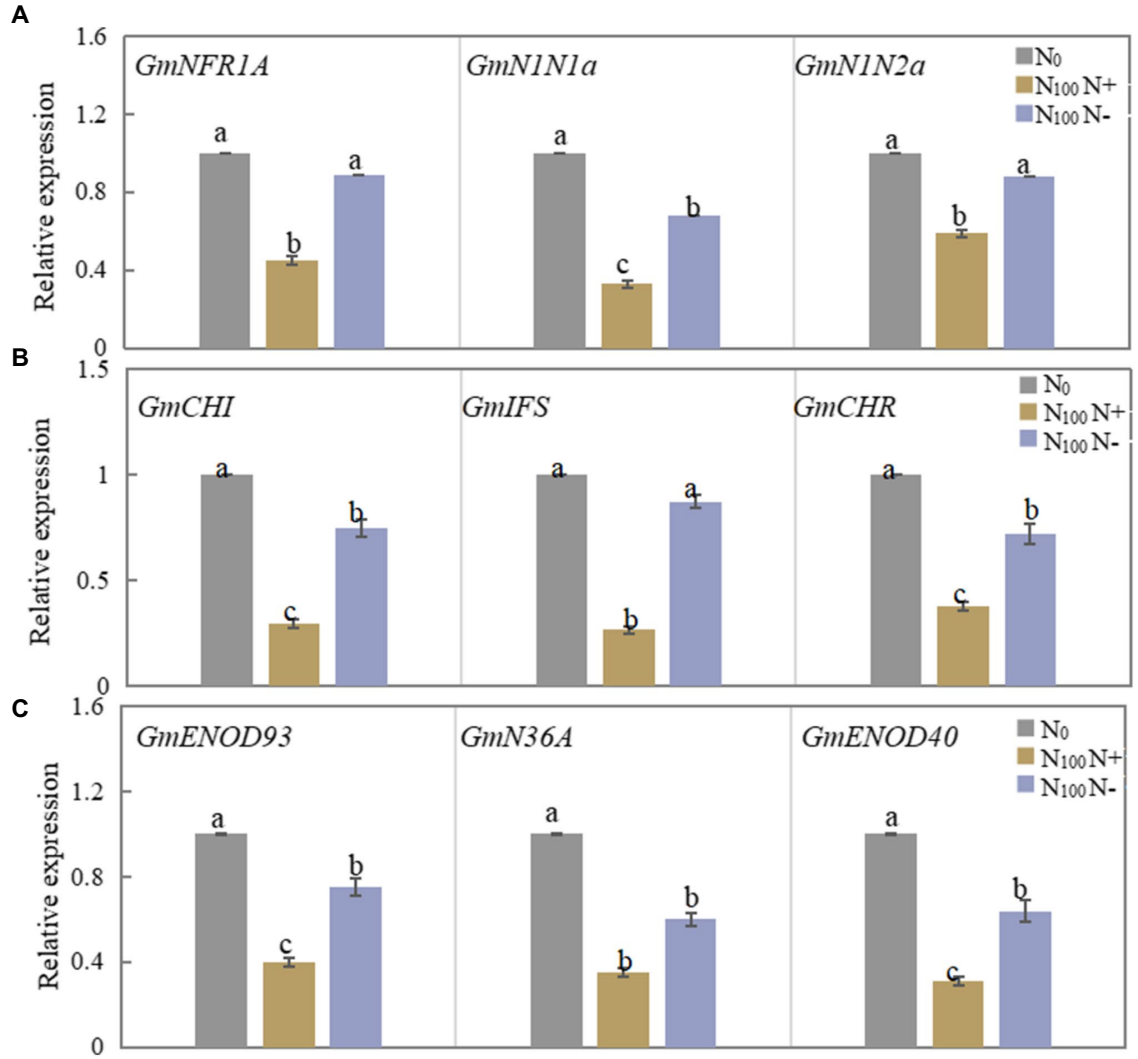
Changes of nodulation-related genes expression in roots and nodules of dual-root soybean, N_0_ is N-free nutrient solution on both sides of dual-root soybeans, N_100_ N+ is N-supply side of dual-root soybeans treated with 100 mg/l nutrient solution on unilateral side, N_100_ N-is N-free side of dual-root soybeans treated with 100 mg/l nutrient solution on unilateral side, different lowercase letters above the bars indicate significant difference (*p* < 0.05) by Duncan’s multiple range test. **(A)** The expression level of the nodulation signal recognition gene on both sides of dual-root soybean after treatment for 3 days. **(B)** The level of expression of the genes for roots isoflavone synthesis on both sides of the dual-root soybean after 7 days of treatment. **(C)** The level of expression of the genes of early nodulin synthesis in root nodules after 21 days of treatment.

As shown in Figure 2A, the relative expression of the *GmNFR1A*, *GmN1N1a* and *GmN1N2a* genes in the roots of N_100_ N+ side after 3 days of N supply was significantly downregulated compared with N_0_ treatment, and the N_100_ N-side also showed a downward trend compared with N_0_, but the reduction was smaller. Figure 2B shows that the expression levels of the three genes in the roots of the N+ side of N100 were significantly reduced, while the expression levels of the three genes in the roots of the N-side of N100 also showed a downward trend, but the reduction was smaller. As shown in Figure 2C, the three genes *GmENOD93*, *GmN36A* and *GmENOD40* all decreased in the nodules on both sides of the N_100_ treatment, while the range of change in the N-free side was smaller than that of the N-supply side. The results showed that the effect of N supply from one side of the dual-root soybean root system on root nodules on both sides of the roots started from the mutual recognition of early nodulation signals, and the regulation of nodulation process also involved changes in the synthesis of isoflavones in the roots.

### Effects of unilateral supply of isoflavones on nitrogen fixation in soybean plants

Table 4 shows the changes in the number and dry weight of the dual-root soybean nodules after the unilateral application of isoflavones. The nodule number and dry weight of the T+ side in the I_3.75_ treatment were significantly higher than those in the I_0_ treatment, increasing by 29.20 and 7.78%, respectively. The nodule number and dry weight of the T-side in the I_3.75_ treatment were significantly lower than those in the I_0_ treatment. This showed that the mixed application of daidzein and genistein improved the nodule number and dry weight on the contact side, but the nodule number and dry weight on the untouched side decreased.

**Table 4:** Effects of unilateral application of isoflavones on double-rooted soybean nodules.

Treatments	Nodule number (per plant)		Nodule dry weight (g/plant)	
	T+	T−	T+	T−
I_0_	113.0 ± 9.71b	113.0 ± 9.71a	0.18 ± 0.033b	0.18 ± 0.033a
I_3.75_	146.0 ± 11.45a	98.6 ± 10.74b	0.23 ± 0.038a	0.12 ± 0.009b

I_0_ is the nutrient solution without isoflavones supplied on both sides, and I_3.75_ is the nutrient solution with daidzein and genistein applied unilateral side. Treated side denoted as T+, untreated side denoted as T–, the values in the table represent the mean ± standard error (*n* = 4), different lowercase letters indicate significant difference (*p* < 0.05) by Duncan’s multiple range test and longitudinal comparison.

As shown in Table 5, there were changes in the SNA and ARA of dual-root soybean after unilateral application of isoflavones. There was no significant difference in SNA on the T+ side of I_0_ and I_3.75_ treatments, while ARA on the T+ side of I_3.75_ treatment was significantly higher than that of I_0_ treatment. T-side SNA in I_3.75_ and I_0_ treatments had no significant difference, but the ARA of I_3.75_ treatment in T-side was significantly lower than that in I_0_ treatment. The mixed application of daidzein and genistein increased the activity of whole plant nitrogenase in nodules on the contact side, but it decreased on the untouched side. There was no significant change in nitrogenase activity per unit weight of nodules. The change of whole plant nitrogenase activity in nodules primarily originated from the change of nodule number and dry weight.

**Table 5:** Effects of unilateral application of isoflavones on nitrogenase activity.

Treatments	SNA(C_2_H_4_ μmol g^−1^ nodule dry mass h^−1^)		ARA(C_2_H_4_ μmol h^−1^ plant^−1^)	
	T+	T−	T+	T−
I_0_	24.5 ± 2.91a	24.5 ± 2.91a	4.4 ± 0.07b	4.4 ± 0.07a
I_3.75_	23.3 ± 2.68a	21.5 ± 0.80a	5.3 ± 0.11a	2.5 ± 0.02b

I_0_ is the nutrient solution without isoflavones supplied on both sides, and I_3.75_ is the nutrient solution with daidzein and genistein applied unilateral side. Treated side denoted as T+, untreated side denoted as T−, the values in the table represent the mean ± standard error (*n* = 4), different lowercase letters indicate significant difference (*p* < 0.05) by Duncan’s multiple range test and longitudinal comparison.

### Effects of isoflavones unilateral supply on isoflavones concentration in soybean roots and root exudates

**Figure 3:**
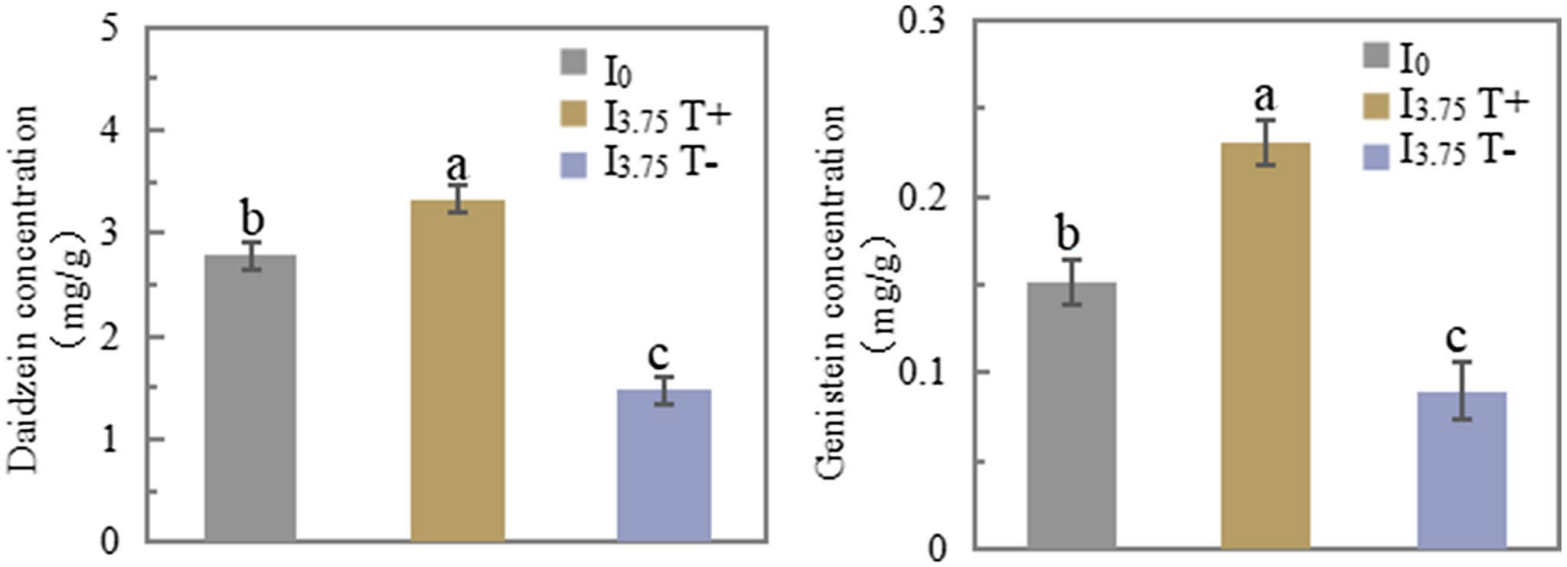
Changes in daidzein and genistein concentrations in roots after unilateral supply of isoflavones, different lowercase letters above the bars indicate significant difference (*p* < 0.05) by Duncan’s multiple range test. I_0_ refers to conventional nutrient solution applied on both sides; I_3.75_ T+ is the treatment side of unilateral application of nutrient solution containing daidzein and genistein, and I_3.75_ T-is the untreated side of the unilateral application of nutrient solution that contained daidzein and genistein.

On 7 days after treatment, the daidzein and genistein concentrations in T+ side roots of the I_3.75_ treatment were significantly higher than those of the I_0_ treatment. The daidzein and genistein concentrations in T-side roots of I_3.75_ treatment were significantly lower than those of I_0_ treatment (Figure 3). The changes of daidzein and genistein concentrations in root exudates were consistent with those in roots (Supplementary Table S4). The mixed application of daidzein and genistein significantly increased the concentrations of daidzein and genistein on the contact side of roots and root exudates, while the concentrations of daidzein and genistein on the untouched side of the roots and root exudates decreased significantly, which was opposite to the trend on the contact side.

### Effects of unilateral isoflavones supply on relative expression of nodulation-related genes

**Figure 4:**
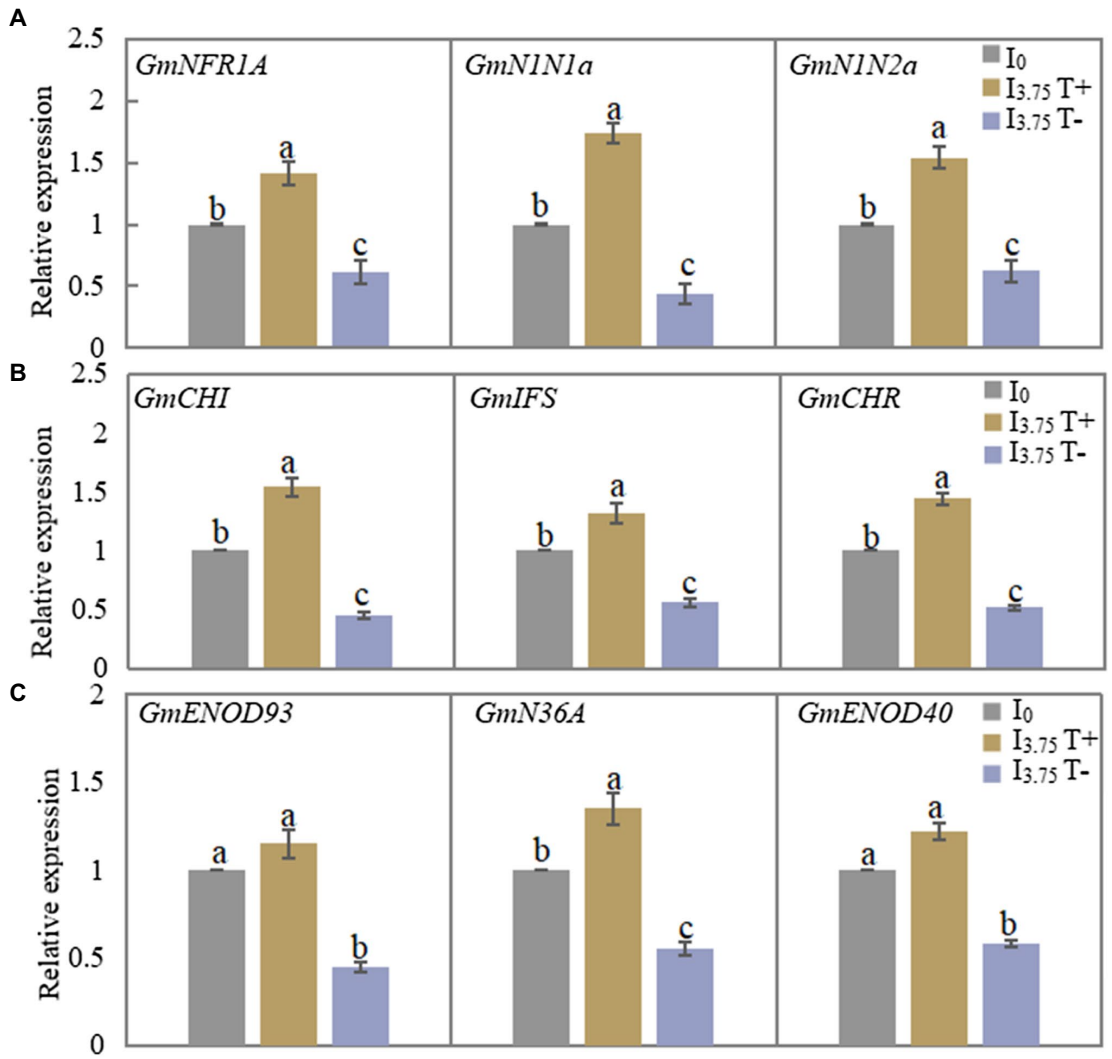
Changes of relative expression of nodulation-related genes in roots and nodules of dual-root soybean after unilateral supply of isoflavones, different lowercase letters above the bars indicate significant difference (*p* < 0.05) by Duncan’s multiple range test. I_0_ refers to conventional nutrient solution applied on both sides; I_3.75_ T+ is the treatment side of unilateral application of nutrient solution containing daidzein and genistein, and I_3.75_ T-is the untreated side of the unilateral application of nutrient solution that contained daidzein and genistein. **(A)** The expression level of the nodulation signal recognition gene on both sides of dual-root soybean after treatment for 3 days. **(B)** The level of expression of the genes for roots isoflavone synthesis on both sides of the dual-root soybean after 7 days of treatment. **(C)** The level of expression of the genes of early nodulin synthesis in root nodules after 21 days of treatment.

As shown in Figure 4A, the relative expressions of the three genes involved in the recognition of early nodulation signals increased significantly after the mixed administration of two isoflavones on the T+ side of I_3.75_ treatment, while the expression levels of the three genes on the T-side significantly decreased. Figure 4B shows that the relative expression levels of the three genes involved in the synthesis of isoflavones on the T+ side increased, while the expression levels of the T-side genes decreased in I_3.75_ treatment. As shown in Figure 4C, the expression of *GmENOD93* and *GmENOD40* on the T+ side of I_3.75_ treatment was higher than that of I_0_ treatment, but the difference was not significant, while the relative expression of *GmN36A* gene is significantly higher than that of I_0_ treatment, I_3.75_ treatment T-side three genes relative expression was significantly decreased. Additionally, the unilateral application of isoflavones affected the signal recognition of nodules, isoflavone synthesis and the growth process of nodules.

## Discussion

### Systematic impacts of nitrogen on nitrogen fixation and nodulation in soybeans

**Figure 5:**
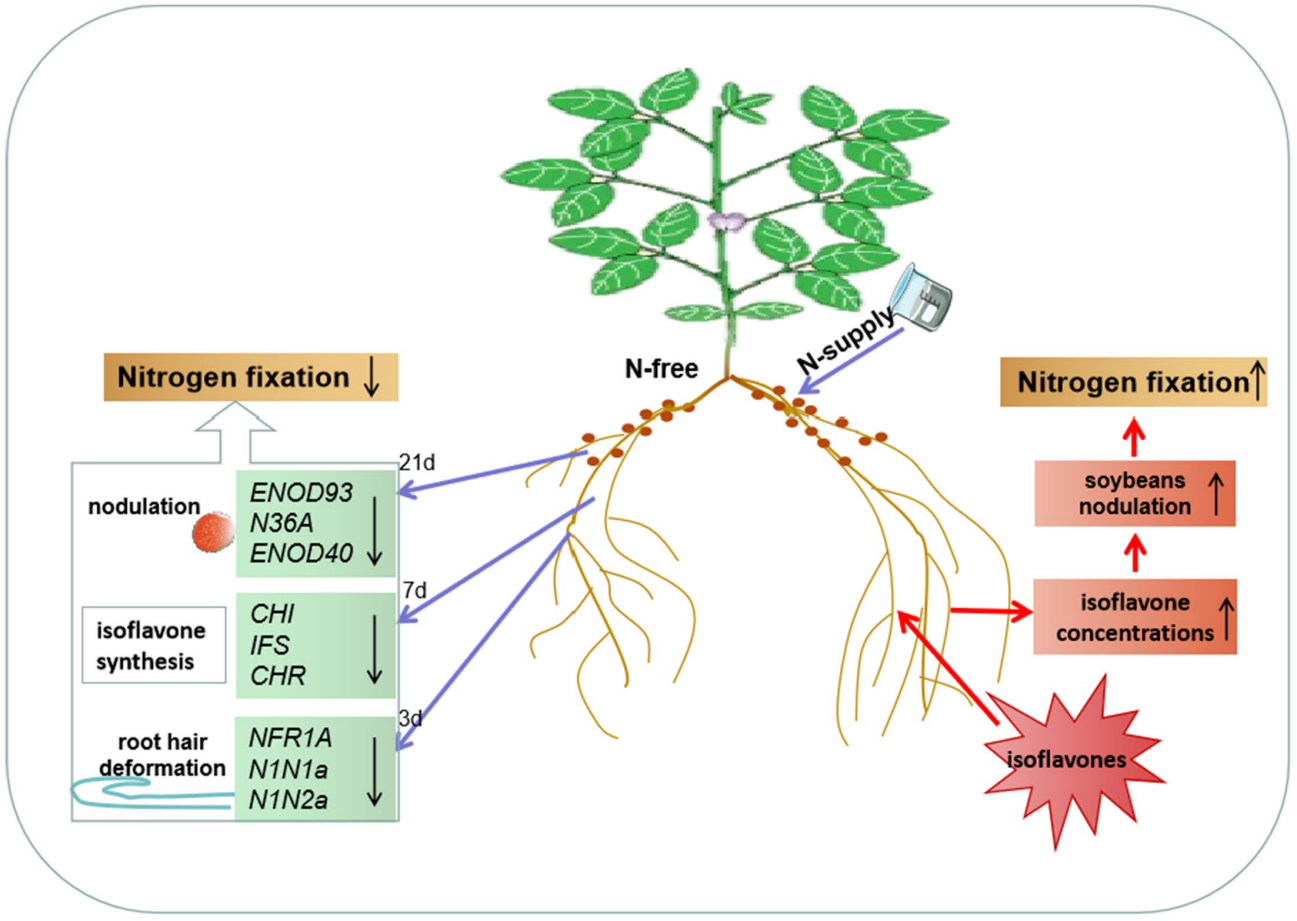
N systemically regulates soybean nodulation and N fixation through isoflavones. After unilateral N-supply, the expression of *NFR1A*, *N1N1a, N1N2a*, *CHI*, *IFS*, *CHR, ENOD93*, *N36A*, and *ENOD40* in the roots and nodules of the N-free side was downregulated, which at least partly explained root hair deformation, nodulation and isoflavone synthesis and secretion enhancement changes. Simultaneous, application of isoflavones promoted the isoflavones synthesis on the treated side and N_2_ fixation in root nodules. Unilateral N or isoflavones supply, nodulation, nitrogen fixation and nodulation-related genes responded to the changes in root isoflavones concentrations in the same way.

The most significant characteristic of infection is the root hair deformation caused during the early stage of infection of the host by rhizobium (Zaat et al., 1987). *NFR1* and *NIN* are involved in signal recognition, infection thread formation and other processes during the early development of root nodules (Schauser et al., 1999; Indrasumunar et al., 2011). The mutation of *NFR1* can prevent early nodulation phenomena, such as root hairs deformation (Amor et al., 2003; Madsen et al., 2003; Radutoiu et al., 2003; Miwa et al., 2006). With the exacerbation of root hair deformation of soybean, the relative expression of *NIN* increased (Wang et al., 2019). A high concentration of N was found to significantly reduce the level of expression of the deformation rate of root hair and *NIN* in *Lotus japonicus* (Barbulova et al., 2007). After 3 days of unilateral N supply in this experiment, root hair deformation on both sides was inhibited. The levels of expression of root *GmNFR1A, GmN1N1a* and *GmN1N2a* decreased significantly on the N+ side and slightly on the N-side (Figure 5). The levels of expression of root hair deformation and early nodulation genes on both sides of soybean were inhibited by the unilateral N supply system, which was similar to the results of previous studies. In addition, the dual-root soybean study revealed that the systematic influence of N on soybean nodules appeared during the early stage of nodulation. Ma et al. (2019) and Zou et al., (2019) reported that the levels of expression of *GmNIN* and *GmNFR1* in soybean roots increased with the increase in N_2_ fixation by nodulation in soybean roots. In this experiment, the nodule number, dry weight, SNA and ARA on both sides decreased after 21 days of unilateral N supply. After N removal, N_2_ fixation on both sides of the soybean recovered. Previous studies have demonstrated that soybean that was supplied with high N on one side of the root inhibited the N_2_ fixation on both sides of the root system (Xia et al., 2017; Lyu et al., 2019; Lyu et al., 2020; Li et al., 2021). *GmENOD93, GmN36A* and *GmENOD40* participate in nodulin synthesis and are closely related to the growth of root nodules (Nguyen et al., 2019; Wang et al., 2019). In this experiment, the relative levels of expression of *GmENOD93, GmN36A* and *GmENOD40* in both sides nodules decreased with the unilateral N supply, which is consistent with the trend of N_2_ fixation by nodules (Figure 5). Unilateral N supply reduced the degree of root hair deformation on both sides, the ability to fix N by nodules and the level of expression of growth genes in early nodulation and nodules, indicating that the unilateral N supply systematically affected the N_2_ fixation in soybean, this effect began during the early stage of nodulation.

### Systematic impacts of nitrogen on the concentration of soybean isoflavones in the roots

Isoflavones secreted by soybean roots can activate the level of expression of nodulation factor nod genes of rhizobium, which play an important role in the development of nodules and their ability to fix N (Maj et al., 2010; Hassan and Mathesius, 2012). Genistein and daidzein are isoflavones that are the most prevalent in soybean (Peters et al., 1986; Kosslak et al., 1987; Smit et al., 1992; Sugiyama et al., 2007). The application of ammonium N, nitrate N and urea significantly reduced the daidzein and genistein concentrations in soybean roots, and the number of nodules decreased (Cho and Harper, 1991). Sugiyama et al. (2016) reported that N supply significantly reduced the daidzein and genistein concentrations in soybean roots. Cho and Harper (1991) supplied N to the unilateral side of split-root soybeans, and nodule number and nitrogenase activity, as well as the daidzein and genistein concentrations in the roots, were lower on the N-supply side than on the N-free side. In this experiment, after the unilateral N supply of dual-root soybean for 7 days, the concentrations of daidzein and genistein in the roots and root exudates on the N-supply side were significantly reduced; the daidzein content on N-free side was significantly reduced, and the concentrations of genistein decreased slightly. After N removal treatment, the concentrations of daidzein and genistein in the roots on both sides of the dual-root soybean increased (Figure 5). The chalcone synthase gene (*GmCHS)* is involved in the synthesis of isoflavone basic skeleton; the isoflavone synthase gene (*GmIFS*) is involved in the synthesis of daidzein and genistein, and the chalcone reductase gene (*GmCHR)* is the key gene for daidzein synthesis (Sugiyama et al., 2016). After silencing the ability of soybean to synthesize key isoflavone genes, soybean inoculated with root nodulation bacteria failed to form root nodules (Subramanian et al., 2006; Wasson et al., 2006; Zhang et al., 2009). In this experiment, the isoflavones synthesized genes (*GmCHI, GmIFS* and *GmCHR*) level of expression on the N supply side roots decreased; the daidzein synthesis genes (*GmCHS* and *GmCHR*) level of expression on the N-free supply side roots decreased significantly, and the genistein synthesis gene (*GmIFS)* level of expression decreased slightly. The trend of changes in the three genes of the roots on both sides was consistent with that of the concentrations of root isoflavones, indicating that the reduction of root isoflavone concentrations caused by the N supply was related to their synthesis. In this experiment, the dual-root soybean unilateral N supply method was used to study the systematic regulation of isoflavone concentrations by N. The trend of changes in the concentrations of root and root exudates isoflavones on 7 days after treatment of the N supply was found to be consistent with that of the nodule numbers and nodulation-related genes after 21 days of treatment in soybeans, and the change in root system on both sides was synchronous. This suggested that soybean nodulation is systematically regulated by N, which is closely related to the early synthesis of isoflavones in the root. The unilateral N supply system inhibits isoflavone synthesis and secretion on both sides of the soybean roots, which affects the recognition process of rhizobium and the root system in the rhizosphere and thus affects soybean nodulation.

As the concentrations of genistein and daidzein in soybean roots decreased, the nodule number and weight decreased (Cho and Harper, 1993; Miransari and Smith, 2007). Rhizobium was used to inoculate the plants after genistein and daidzein culture to improve the dry weight and N_2_ fixation capacity of soybean root nodules (Zhang and Smith, 1995; Miransari et al., 2009; Dolatabadian et al., 2012; Riviezzi et al., 2020). In this study, the mixed application of treatment with two isoflavones increased the concentrations of daidzein and genistein in roots on the T+ side, increased the level of expression of genes involved in isoflavone synthesis (*GmCHI*, *GmIFS* and *GmCHR*), and also increased nodule number and dry weight. It also increased the expression of genes involved in the early nodulation process (*GmNFR1A*, *GmN1N1a* and *GmN1N2a*) and nodule synthesis (*GmENOD93*, *GmN36A* and *GmENOD40*) in root nodules (Figure 5), indicating that the unilateral application of isoflavone not only promoted isoflavone synthesis in roots but also stimulated the expression of early nodulation genes and root nodule growth genes, so as to improve the effect of N_2_ fixation by nodulation in soybean. In this study, under the conditions of unilateral N supply and unilateral external application of isoflavones, there was an identical response of N_2_ fixation by nodulation in soybean and its nodulation-related genes to root isoflavone concentrations, which decreases with the decrease in the concentrations of root and root exudates isoflavones. It was demonstrated that N regulates N_2_ fixation by nodulation in soybean through its systematic impacts on the concentrations of root isoflavones. However, the phenomenon of increased expression of early nodulation genes and root nodule growth genes depends on the exogenous application of isoflavones, the concentrations of root isoflavones or the synergistic effect of the two merits further research.

## Conclusion

The systemic inhibitory effect of N on N_2_ fixation in soybean nodules began at the early stage of nodulation and systematically inhibited the concentrations of isoflavones in soybean roots and root exudates. The changes in N_2_ fixation by soybean nodules following treatment with a unilateral N supply or isoflavones were consistent with the changes of isoflavone concentrations in the roots and root exudates.

## Data availability statement

The original contributions presented in the study are included in the article/Supplementary material; further inquiries can be directed to the corresponding authors.

## Author contributions

XL and ZG: conceptualization, methodology, and writing—reviewing and editing. CS: data curation. TL and XW: funding acquisition. SL and ZW: investigation. XL and CY: resources. CM: software. XL: writing—original draft. All authors contributed to the article and approved the submitted version.

## Funding

We are grateful for the support from the Project funded by China Postdoctoral Science Foundation (NO. 2022M710651).

## Conflict of interest

The authors declare that the research was conducted in the absence of any commercial or financial relationships that could be construed as a potential conflict of interest.

## Publisher’s note

All claims expressed in this article are solely those of the authors and do not necessarily represent those of their affiliated organizations, or those of the publisher, the editors and the reviewers. Any product that may be evaluated in this article, or claim that may be made by its manufacturer, is not guaranteed or endorsed by the publisher.
