# Supplementary material for: Systemic regulation of soybean nodulation and nitrogen fixation by nitrogen via isoflavones
Source: Front Plant Sci. 2022 Aug 11;13:968496. doi: 10.3389/fpls.2022.968496 (PMC9403732; doi:10.3389/fpls.2022.968496)
Supplement: Supplementary file 1 [file Data_Sheet_1.docx]

Supplementary Material


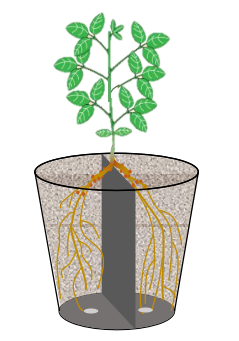


**Supplementary Figure 1.** Pot plot of dual-root soybeans. The diameter of each plastic bucket was 0.30 m, and the height was 0.28 m. The plastic bucket was divided by a plastic board that fit the inside the bucket into two equal parts to allow two roots to grow separately. 1-cm-diameter drain holes were drilled at the bottom of the barrel. The seedlings were treated with 250 mL of distilled water on each side once a day before the opposite true leaves were fully expanded; 250 mL of nutrient solution was administered on both sides once a day after the opposite true leaves were expanded. When the true soybean leaves were completely unfolded, the field soybean nodules cryopreserved in the previous year were ground and added to the nutrient solution, with approximately 5 g of nodules per liter, and inoculated for 5 consecutive days.


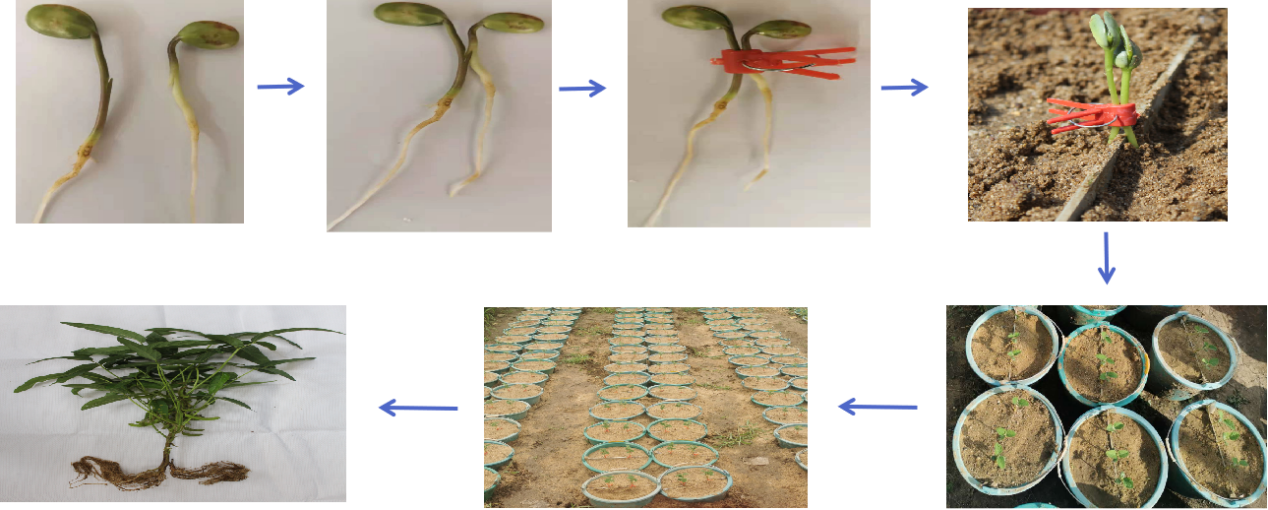


**Supplementary Figure 2.** Preparation of dual-root soybean plants material. Seeds were seeded into fine-sand medium and were cultured in a growth chamber at 30°C for approximately 3 days. When the soybean cotyledon grows to 7-10 cm from the root tip, flush out the root system of the seedling with distilled water for grafting. Use a sterilized blade to make a 0.5-1.0 cm long gap upward or downward on the upper middle of the hypocotyl of the two seedlings. Two seedlings were cross-inserted into the cuts of each other. Two seedlings were separately planted into fine-sand medium of each half of the pot divided by the partition plate.


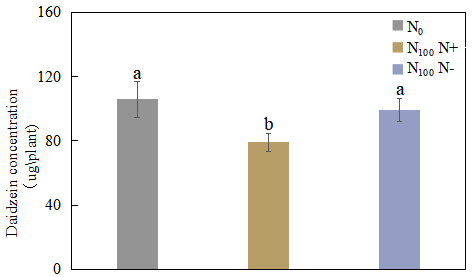

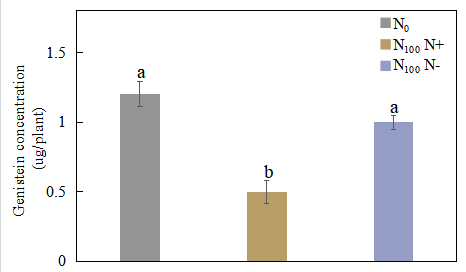


**Supplementary Figure 3.** Changes of daidzein and genistein concentrations in root exudates after unilateral supply of nitrogen 7 days. Different lowercase letters above the bars indicate signifificant difference (*p*< 0.05) by Duncan’s multiple range test. N_0_ is N-free nutrient solution on both sides of dual-root soybeans, N_100_ N+ is N-supply side of dual-root soybeans treated with 100 mg/L nutrient solution on unilateral side, N_100_ N- is N-free side of dual-root soybeans treated with 100 mg/L nutrient solution on unilateral side.


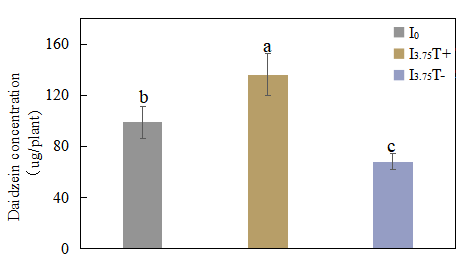

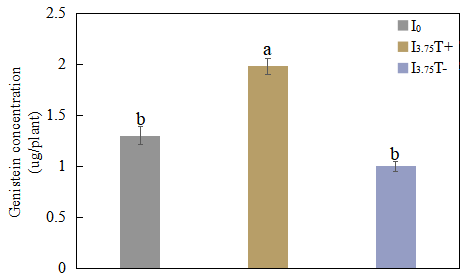


**Supplementary Figure 4.** Changes of daidzein and genistein concentrations in root exudates after unilateral supply of isoflavones 7 days. Different lowercase letters above the bars indicate signifificant difference (*p*< 0.05) by Duncan’s multiple range test. I_0_ refers to conventional nutrient solution applied on both sides; I_3.75_ T+ is the treatment side of unilateral application of nutrient solution containing daidzein and genistein, and I_3.75_ T- is the untreated side of the unilateral application of nutrient solution that contained daidzein and genistein.

**Supplementary Table 1**. Recipe of the universal nutrient solution for sand culture

| Inorganic salts | Concentration (mg/L) | Inorganic salts | Concentration (mg/L) |
| --- | --- | --- | --- |
| KH_2_PO_4_ | 136.00 | ZnSO_4_·7H_2_O | 0.22 |
| MgSO_4_ | 240.00 | MnCl_2_·4H_2_O | 4.90 |
| CaCl_2_ | 220.00 | H_3_BO_3_ | 2.86 |
| Na_2_MoO_4_·H_2_O | 0.03 | Fe–EDTA | * |
| CuSO_4_·5H_2_O | 0.08 |  |  |

*indicates that the solution which contains 5.57 g FeSO_4_·7H_2_O and 7.45 g Na_2_EDTA per one L, respectively, was added into nutrient medium as the rate of 1:1000 when using. The experiment of nitrogen or isoflavone supply treatment is based on the addition of NH_4_NO_3_ or daidzein and genistein to the nutrient solution.

**Supplementary Table 2**. Primer sequence of related genes

| Genes | Primer sequence |
| --- | --- |
| *GmNFR1A*-F | 5’-AACAGGTCTTGCTAGGGGTG-3’ |
| *GmNFR1A*-R | 5’-GACCGTACCATCTTGAGTTGA-3’ |
| *GmNIN1a*-F | 5’-TGGCGCACCATGCTAACAT-3’ |
| *GmNIN1a*-R | 5’-GGGTGTCATGGCAATCCTTT-3’ |
| *GmNIN2a*-F | 5’-CAAGCCCTGTTTTGCTATGGA-3’ |
| *GmNIN2a*-R | 5’-TAGAGGAATAGCGACGGCAGAA -3’ |
| *GmCHS*-F | 5’-TTGAGACTGTTGTGCTCCGC-3’ |
| *GmCHS*-R | 5’-AAGCATGTGAGGGAAGCAGGG-3’ |
| *GmCHR*-F | 5’-TCCACTGATGCTCATCCTGAC-3’ |
| *GmCHR*-R | 5’-TTCCATAGCCTCCCATATTCC-3’ |
| *GmIFS-*F | 5’-GACAGACCATCAGAATTCCGTC-3’ |
| *GmIFS*-R | 5’-CTAACTTTGGCATCATCACCTT-3’ |
| *GmENOD93*-F | 5’-GCCTCTGCCATTCCAACTCT-3’ |
| *GmENOD93*-R | 5’-AAGTATGCTGCTGCCGTCG-3’ |
| *GmN-36A*-F | 5’-GATAAAGGGGTGTGAGAGGGTC-3’ |
| *GmN-36A*-R | 5’-CTGCCACTCAAGAGAGAATGTTAGA-3’ |
| *GmENOD40*-F | 5’-TCTCTCTTGAGTGGCAGAAGCA-3’ |
| *GmENOD40*-R | 5’-TGGAGTCCATTGCCTTTTCG-3’ |
| *Gm18s*-F | 5’-CCATAAACGATGCCGACCAG-3’ |
| *Gm18s*-R | 5’-AGCCTTGCGACCATACTCCC-3’ |
